# Circumstances and factors associated with accidental deaths among children, adolescents and young adults in Cuiabá, Brazil

**DOI:** 10.1590/1516-3180.2013.1314459

**Published:** 2013-08-01

**Authors:** Christine Baccarat de Godoy Martins, Maria Helena Prado de Mello-Jorge

**Affiliations:** I PhD. Associate Professor, School of Nursing, Universidade Federal de Mato Grosso (UFMT), Cuiabá, Mato Grosso, Brazil.; II PhD. Associate Professor, School of Public Health, Universidade de São Paulo (USP), São Paulo, Brazil.

**Keywords:** Accidents, Child, Risk factors, Mortality, Epidemiology, Acidentes, Criança, Fatores de risco, Mortalidade, Epidemiologia

## Abstract

**CONTEXT AND OBJECTIVE:**

Analysis on accidents from the perspective of population segments shows there is higher incidence among children, adolescents and young adults. Since the characteristics and circunstances of the event are closely related to educational, economic, social and cultural issues, identifying them may contribute towards minimizing the causes, which are often fatal. The aim here was to identify the environmental, chemical, biological and cultural factors associated with deaths due to accidents among children, adolescents and young adults in Cuiabá, in 2009.

**DESIGN AND SETTING:**

This was a descriptive cross-sectional study conducted in Cuiabá, Mato Grosso, Brazil.

**RESULTS:**

Thirty-nine accidental deaths of individuals aged 0 to 24 years were examined: 56.4% due to traffic accidents; 25.6%, drowning; 10.3%, aspiration of milk; 5.1%, falls; and 2.6%, accidentally triggering a firearm. Male victims predominated (82.1%). The presence of chemical, environmental and biological risk factors was observed in almost all of the homes. Regarding cultural factors and habits, a large proportion of the families had no idea whether accidents were foreseeable events and others did not believe that the family’s habits might favor their occurrence. Delegation of household chores or care of younger siblings to children under the age of 10 was common among the families studied.

**CONCLUSION:**

The results point towards the need to have safe and healthy behavioral patterns and environments, and to monitor occurrences of accidents, thereby structuring and consolidating the attendance provided for victims.

## RESUMO

**CONTEXTO E OBJETIVO:**

Ao analisar os acidentes sob o prisma dos segmentos populacionais, observa-se grande incidência em crianças, adolescentes e jovens. A frequência e as características e circunstâncias do evento estão intimamente relacionadas com fatores educacionais, econômicos, sociais e culturais, e a identificação desses fatores pode contribuir para minimizar essas causas, muitas vezes fatais. O objetivo foi identificar os fatores ambientais, químicos, biológicos e culturais associados com óbitos por acidentes, ocorridos entre crianças, adolescentes e jovens em Cuiabá, em 2009.

**TIPO DE ESTUDO E LOCAL:**

Este é um estudo descritivo, transversal, realizado em Cuiabá, Mato Grosso, Brasil.

**RESULTADOS:**

Foram analisados 39 óbitos acidentais ocorridos de 0 a 24 anos (56,4% por acidente de transporte, 25,6% por afogamento, 10,3% por aspiração de leite, 5,1% por queda e 2,6% por disparo acidental de arma de fogo). Houve predomínio no sexo masculino (82,1%). Observou-se a presença de fatores químicos, ambientais e biológicos na quase totalidade das residências. Quanto aos fatores culturais e hábitos, grande parte das famílias não soube referir se o acidente constitui evento previsível e outra parte não acredita que os hábitos da família favorecem sua ocorrência. Delegar aos filhos menores de 10 anos os afazeres domésticos ou o cuidado de irmãos menores é comum entre as famílias estudadas.

**CONCLUSÃO:**

Os resultados apontam para a necessidade de adotar comportamentos e ambientes seguros e saudáveis, bem como monitorizar a ocorrência dos acidentes, estruturando e consolidando o atendimento às vítimas.

## INTRODUCTION

Unintentional events (accidents) give rise to direct and indirect costs. The former consists of expenditure on medical attention and treatment, complementary examinations, hospitalization and rehabilitation; the latter relates to the loss of working days, lower productivity and material damage.^1^

The highest incidence of accidents has been recorded among children, adolescents and young adults, with important peculiarities regarding frequency and the characteristics and circumstances of the event.^2^ Worldwide, the main causes of death during childhood include traffic accidents (mortality rate of 10.7/100,000), drowning (mortality rate of 7.2/100,000), burns (mortality rate of 3.9/100,000), falls (mortality rate of 1.9/100,000) and poisoning (mortality rate of 1.8/100,000).^3^ In Brazil, the deaths among children under ten years of age are due to traffic accidents (29.3%), drowning (21.1%), suffocation (15.4%), aggression (7.0%) and falls (5.1%). In 2009, the mortality rate from traffic accidents for this age group was 3.6/100,000, followed by drowning (2.6/100,000) and accidental risks to breathing (1.8/100,000).^4^

Accidents in the infant-juvenile group stand out not only because of the deaths that they cause, but also because of the resultant trauma, which leaves sequelae and drastically interrupts the growth and development phase.^5^

Some authors have suggested that accidents are closely related to educational, economic, social and cultural factors, such as low income, low maternal education, inadequate spaces for leisure, poor facilitating physical structure within the environments, high levels of street exposure, inadequate supervision, dysfunctional family constitution, family conflicts and consumption of alcohol and drugs, among others.^3^^,^^6^^,^^7^ Therefore, identification and reduction of such factors may contribute towards reducing accidents and, consequently, these traumatic situations and their often irreversible or fatal consequences.

## OBJECTIVE

The present study aimed to identify environmental, chemical, social and cultural factors present in the homes of children, adolescents and young adults who died in an accident.

## METHODS

This was an epidemiological investigation in which the study population comprised children, adolescents and young adults (0 to 24 years of age) who lived in Cuiabá, Mato Grosso, Brazil, and died in accidents between January 1 and December 31, 2009. All the accidental deaths among individuals aged 0 to 24 years that occurred in the municipality in 2009 were studied, thus including the whole population and not just a sample.

Identification data for the victims were obtained from their death certificates, which were available by the Births and Deaths Registry of the Municipal Health Department of Cuiabá. The inclusion criteria were as follows: age 0 to 24 years; resident in Cuiabá; death occurred in 2009; and accidental cause was the basic cause of death (chapter 20 of ICD-10; codes V01 to X59).

A domestic investigation among the victims’ families was then performed using a form with closed questions.

The data were analyzed using the EpiInfo software, with simple and bivariate analyses, and P < 0.005 was taken to be the significance level.

The variables studied were: type of accident (subgroups) according to age and sex of the victims; circumstances of death; place of the accident; place of death; period of the day (morning, afternoon or evening) and day of the week (beginning, middle or end of the week) on which the accident occurred; who the victim was with at the time of the accident; and factors observed during the domestic survey (environmental, chemical, biological and cultural factors).

The “environmental factors” considered were: stairs, verandas, access ramps, windows and swimming pools without protection; furniture at windows; sharp pointed devices, tools, plastic bags, matches and lighters within reach of children; access to the bathroom, laundry, kitchen, stove and hot products; stove in an area without ventilation; cookware with handles projecting out from the stove or with lids that did not fit; tablecloths with corners that could be pulled; access to electrical wiring and sockets; glassware and cans in low places; toys and/or objects scattered on the floor; loose rugs; wet floors; buckets, basins or bath with water; objects on the stairs; piles of firewood, tiles, bricks or planks; garbage bins without lids; open water tank or cesspool; access to machinery or equipment; wire fences; hammocks suspended at 1.0-1.5 m from the ground; fans with the propellers exposed; uneven floors; slippery floors; or firearms in the home.

The “chemical factors” considered were: cleaning products (detergent, soap and sanitary water); volatile chemicals (alcohol, kerosene and gasoline); solvents (thinner); poisons; cosmetics (cream, nail polish, make-up and perfume); hygiene products (soap, shampoo and deodorant); and medicines within the reach of children, according to the way in which they are stored and accessed.

The “biological factors” considered were: presence of animals, a garden with tall grass, plants within the reach of children and trees in the yard.

The “cultural factors” considered were: the family’s perception of predictability of the event; the family’s beliefs about habits and lifestyle favoring accidents; the family’s supervision of the child while in water (swimming pool, bucket, tank or river) or at leisure; use of a baby walker; the habit of leaving the child alone on a bed, sofa or diaper change table; the victim’s habit of playing on stairs, roofs or verandas, or flying a kite near electric wires; and the habit of delegating household chores or the task of caring for small children to children under ten years of age.

The mortality rate per accident was calculated based on the population of the same age and year.

This research was authorized by the Health Department of Cuiabá and the Research Ethics Committee of the University Hospital Julius Müller University Hospital (Hospital Universitário Júlio Müller, HUJM) of the Federal University of Mato Grosso (Universidade Federal de Mato Grosso, UFMT) (protocol 929/CEP-HUJM/2010). The participating families signed a free and informed consent statement.

## RESULTS

This study included 39 cases of death due to accidental causes among children, adolescents and young adults aged 0 to 24 years of age, who were living in Cuiabá, Mato Grosso, Brazil, in 2009. Regarding the accidents, 56.4% involved traffic accidents, followed by drowning (25.6%), risks relating to breathing/aspirating milk (10.3%), falls (5.1%) and accidental triggering of firearm (2.6%). Regarding gender, males predominated (82.1%) (Table 1).

**Table 1.** Distribution of deaths due to accidental causes among children, adolescents and young adults, according to kind of accident, age and gender, Cuiabá, 2009

Accidental cause (subgroup) and proportion (%) in relation to total deaths	Victim’s age (in years)	n (f)	(%) in relation to the subgroup of cause	Frequency by gender
Ground transportation accident (22) 56.4%	10 to 14	2	9.1	(1) Male (1) Female
	15 to 19	9	40.9	(8) Male (1) Female
	20 to 24	11	50.0	(10) Male (1) Female
Drowning (10) 25.6%	1 to 4	2	20.0	(2) Male
	5 to 9	1	10.0	(1) Male
	15 to 19	2	20.0	(1) Male (1) Female
	20 to 24	5	50.0	(5) Male
Accidental breathing risk (4) 10.3%	< 1 year	4	100.0	(2) Male (2) Female
Fall (2) 5.1%	15 to 19	2	100.0	(2) Male
Inanimate mechanical force (1) 2.6%	5 to 9	1	100.0	(1) Female
Total deaths		39	100.0	Male = 32 (82.1%) Female = 7 (17.9%)

Transportation accidents occurred at a higher rate in the age group from 20 to 24 years (50.0%), as did drowning (drowning in rivers), although this was also present in the age groups from one to four years (drowning in pools and lakes) and from five to 9 (drowning in lakes). The cases of asphyxia (aspiration of milk) occurred most often among children under one year of age, falls in the age group from 15 to 19 years (from scaffolding during work) and accidental triggering of a firearm in the age group from five to nine years, while playing with the father’s weapon (Table 1).

**Figure 1.**
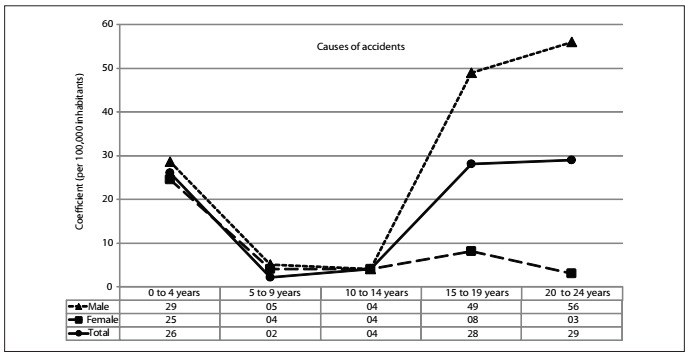
Mortality coefficient from cause of accidents among children, adolescents and young adults, according to age group and gender, Cuiabá, 2009.

The mortality coefficient from accidents, calculated on the basis of the population and year of this study, showed higher mortality rates among males, especially after the age of 15 years (Figure 1).

Most victims of accidents died in hospital. Among the victims whose accidents occurred at home, 85.7% died in hospital and the remainder (14.3%) died at the location of the event (home). Among the victims whose accidents occurred on public thoroughfares (in the case of transport accidents), 57.1% died in hospital and 42.9% at the location of the accident. The workplace accidents also led to death in hospital.

Transportation accidents occurred predominantly in the morning (54.5%) and in the middle of the week (59.1%), whereas drowning occurred in the evening (90.0%) and on the weekend (80.0%). The cases of asphyxia (aspiration of milk) occurred in the morning and evening, falls in the morning and accidents with firearms in the afternoon. These last three events occurred in the middle of the week (Table 2).

**Table 2.** Distribution of deaths due to accidental causes among children, adolescents and young adults, according to kind of accident, part of the day and part of the week in which they occurred, Cuiabá, 2009

Accidental cause	Part of the day in which the event occurred										P-value
	Morning		Afternoon		Evening		Night		Total		
	n	%	n	%	n	%	n	%	n	%	
Transport accident	12	54.5	3	13.6	3	13.6	4	18.2	22	100.0	
Drowning	1	10.0	9	90.0	-	-	-	-	10	100.0	
Milk aspiration	2	50.0	-	-	2	50.0	-	-	4	100.0	
Fall	2	100.0	-	-	-	-	-	-	2	100.0	
Accidental triggering of firearm	-	-	1	100.0	-	-	-	-	1	100.0	
Total	17	43.6	13	33.3	5	12.8	4	10.3	39	100.0	0.0002
Accidental cause	Part of the week in which the event occurred								P-value
	Beginning of the week		Middle of the week		End of the week		Total		
	n	%	n	%	n	%	n	%	
Transport accident	2	9.1	13	59.1	7	31.8	22	100.0	
Drowning	1	10.0	1	10.0	8	80.0	10	100.0	
Milk aspiration	-	-	4	100.0	-	-	4	100.0	
Fall	-	-	2	100.0	-	-	2	100.0	
Accidental triggering of firearm	-	-	1	100.0	-	-	1	100.0	
Total	3	7.7	21	53.8	15	38.5	39	100.0	0.0016

At the time of the accident, 68.2% of the victims of traffic accidents were alone and 22.0% were with their parents, although all the victims were the drivers. In the cases of drowning, 70.0% of the victims were also alone and only 30.0% were in the company of their parents. The children who died from aspiration of milk were with their parents; the adolescents who died due to falls were alone; and the child who died from accidental triggering of a firearm was in the company of his grandfather while the parents were working.

Regarding environmental factors, most of the homes showed the risk factors mentioned in this study, with the exception of firearms (Table 3). These variables were listed in order to determine the frequency of each risk factor in the 39 houses studied. The findings show that none of the homes was absolutely risk-free. Sharp-pointed materials, tools, plastic bags and matches were observed to be within the reach of children in all the homes, and the children had free access to the kitchen, bathroom, laundry and stove. The only homes that did not present open water tanks or cesspools were the very ones that did not possess either a water tank or a cesspool.

**Table 3.** Distribution of deaths due to accidental causes among children, adolescents and young adults, according to environmental factors observed in the victims’ homes (n = 39), Cuiabá, 2009

Environmental factors observed in the victims’ homes	Yes		No	
	n	%	n	%
Sharp-pointed devices within children’s reach	39	100.0	-	-
Tools within children’s reach	39	100.0	-	-
Plastic bags within children’s reach	39	100.0	-	-
Matches/lighters within children’s reach	39	100.0	-	-
Access to bathroom	39	100.0	-	-
Access to laundry	39	100.0	-	-
Access to kitchen	39	100.0	-	-
Access to stove	39	100.0	-	-
Access to hot products	39	100.0	-	-
Verandas without protection	39	100.0	-	-
Access ramps without protection	37	94.9	2	5.1
Piles of firewood, tiles, bricks or planks in the backyard	37	94.9	2	5.1
Fan with exposed blades	37	94.9	2	5.1
Furniture at the window	35	89.7	4	10.3
Cookware with handle projecting out from stove	35	89.7	4	10.3
Garbage bins without lid	35	89.7	4	10.3
Access to electric wiring/sockets	35	89.7	4	10.3
Tablecloths with long tips	34	87.2	5	12.8
Buckets/basins/bath containing water	34	87.2	5	12.8
Objects on the stairs	34	87.2	5	12.8
Toys and objects scattered over the floor	33	84.6	6	15.4
Loose rugs	33	84.6	6	15.4
Hammocks suspended at 1.0 to 1.5 m from the ground	33	85.0	6	15.0
Windows without protection	31	74.5	8	20.5
Wet floors	31	74.5	8	20.5
Slippery floors	31	74.5	8	20.5
Stove in area without ventilation	31	74.5	8	20.5
Pans with lids that do not fit	27	69.2	12	30.8
Glassware and cans within children’s reach	27	69.2	12	30.8
Staircases with protection	27	69.2	12*	30.8*
Access to machinery/equipment	24	61.5	15	38.5
Uneven floor	24	61.5	15	38.5
Swimming pool without protection	8	20.5	31^†^	79.5^†^
Open water tank	7	17.9	32^‡^	82.1^‡^
Wire fences	4	10.3	35	89.7
Open cesspool	2	5.1	37^‡^	94.9^‡^
Firearm at home	1	2.6	38	97.4

*There were no stairs in the home. ^†^There was no swimming pool in the home; ^‡^There was no water tank/cesspool in the home.

In most of the homes, chemical factors were in easy reach of children and many of these products were even packed in food jars or bottles (Table 4).

**Table 4.** Distribution of deaths (n = 39) due to accidental causes among children, adolescents and young adults, according to chemical and biological factors observed in the victim’s home and cultural factors and habits of the victim’s family, Cuiabá, 2009

	Yes		No	
	n	%	n	%
Chemical factors observed in the victim’s home				
Cleaning products within children’s reach	39	100.0	-	-
Solvents within children’s reach	39	100.0	-	-
Poison within children’s reach	39	100.0	-	-
Cosmetics within children’s reach	39	100.0	-	-
Hygiene products within children’s reach	39	100.0	-	-
Medicines within children’s reach	39	100.0	-	-
Cleaning product in food bottle	33	84.6	6	15.4
Poison in food bottle	31	79.5	8	20.5
Volatile products within children’s reach	26	66.7	13	33.3
Solvent in food bottle	27	69.2	12	30.8
Volatile product in food bottle	19	48.7	20	51.3
Biological factors observed in the victim’s homes				
Animals	32	82.1	7	17.9
Plants within children’s reach	19	48.7	20	51.3
Tall grass	17	43.6	22	56.4
Tree in yard	3	7.7	36	92.3
Family habits and cultural factors				
Children play on roof/stair/veranda	39	100.0	-	-
Children run kites in the street (under electric wiring)	38	97.4	1	2.6
Parents delegate household chores to children < 10 years	38	97.4	1	2.6
Parents delegate the care for younger siblings to child	38	97.4	1	2.6
Parents usually supervise the child in the water	30	76.9	9	23.0
Parents usually use a walker for small children	27	69.2	12	30.8
Family habits favor accidents	20	51.3	19	48.7
Parents usually leave child alone on bed/sofa/diaper changing table	17	43.6	22	56.4
Parents usually supervise the child during leisure activities	10	25.6	29	74.4
Accidents are foreseeable and preventable	3	7.7	36	92.3

Regarding biological factors, the presence of animals (82.1%), trees in the yard (92.3%) and plants and tall grass was common to most homes (Table 4).

Regarding cultural factors and the families’ habits, most of the participants thought that accidents were neither foreseeable events nor preventable (92.3%), while only 7.7% believed that they were so. When inquired whether the family’s habits favored occurrences of accidents, 51.3% said that they did (Table 4).

Table 4 also shows that many families were in the habit of supervising children and adolescents in the water (76.9%) but not during leisure time (74.4%). Moreover, playing on roofs and running a kite in the street were common activities among the children. It was also common for adults to delegate household chores or the care of younger siblings to children under ten years of age.

## DISCUSSION

Among the various types of accidents, the concentration of victims among males, from transport accidents and at younger ages coincides with the results from other studies on causes of accidents.^8^^,^^9^ In view of this context, it has been suggested that legal measures (relating to implementation and enforcement), in association with preventive measures and traffic education, as practiced in developed countries, are essential.^10^

Our findings draw attention to occurrences of drowning in rivers and lakes, both at very young and at older ages. These cases may be explained by the great number of natural water areas in the region of Cuiabá, which are commonly used for leisure in view of the hot climate. Whereas in rich countries drowning occurs predominantly in swimming pools, in poorer countries this type of accident occurs mostly in rivers and public natural water areas.^11^ In view of the high lethality rate due to drowning, it is essential to implement surveillance of children and adolescents during leisure activities in water, as well as providing signs and infrastructure enhancements, with fences surrounding risk areas and the presence of lifeguards. Nevertheless, the vast hydrographic network with many waterfalls and natural water areas makes it difficult to provide lifeguards and surveillance, thus showing the importance of behavioral changes among families, such that safe behavior and surveillance of children become habits.

It is important to highlight the deaths resulting from aspiration of milk, which have also been mentioned in other studies as common among children under the age of one year.^12^ This points towards the need for proper eructation after each breastfeeding session and proper positioning in the cradle to prevent suffocation in the event of regurgitation.^13^

Falls while working, in turn, draw attention to the issue of safety in the workplace. Statistics show that accidents in the workplace are one of the main causes of occupational death throughout the world, although with different coefficients for economically developed countries (5.9 deaths per 100,000 workers), Asian countries (23.1 per 100,000) and Latin American countries (13.5 per 100,000).^14^ In Brazil, the estimated coefficient is 11.4 per 100,000 workers,^15^ which is much higher than in developed countries such as England (0.7 per 100,000).^16^ However, these statistics relate to regular workers. In our study, the deaths from falls in the workplace involved children under the age of 15 years, who are considered under Brazilian law to be apprentices.^17^ Nonetheless, given that accidents in the workplace have a major impact on the productive and economic capacity of the country, since they generally involve young people at the beginning of their professional lives, reflection is required regarding the importance of prevention and safety measures and the use of appropriate equipment, in order to avoid this kind of accident and the consequent injuries, which are often fatal.

Accidental death caused by triggering firearms raises the discussion about the danger of keeping firearms at home, since children and adolescents have natural curiosity and lack of perception of imminent risk form a dangerous combination.^18^ Therefore, it is better not to have a firearm at home; and if this is really necessary, keeping it unloaded and out of reach of children/adolescents are important preventive measures.

With regard to the place of death, the fact that many transport accidents occurred on public thoroughfares and that the victims died at the location of the accident is indicative of the severity of these traffic accidents and raises three important issues. Firstly, in relation to safety equipment that may reduce the severity of injuries, seat belts should be used in automotive vehicles and helmets should be used by drivers and passengers of motorcycles: these are mandatory devices according to the new Brazilian National Traffic Code.^19^ Furthermore, children should always use the correct child restraint such as a child car seat or seat belt, and should travel in the rear seats.^20^ Many deaths can be avoided through correct use of such equipment. However, not all drivers and passengers are using this equipment.^21^

The second issue relates to speedy and skillful care soon after the accident, which is fundamental for reducing the injuries and improving the chances of survival.^10^

Finally, the third issue relates to the need to study the places in which the most serious accidents (with immediate fatal outcome) occur, since these places can be considered to present risks and require careful planning and study. Studying accidents according to their region of incidence (using geographical information systems, GIS) is now capable of generating effective solutions, particularly in relation to traffic accidents.^22^ In healthcare, georeferencing has been highlighted as essential for evaluation and assessment of risks, especially considering that injuries due to traffic accidents (i.e. those responsible for deaths at the site of the accident, in the present study) are responsible for 23% of deaths due to external causes worldwide.^23^

The period of occurrence of transportation accidents in the present study contradicts the findings of most other research, which indicated that greater numbers of traffic accidents occur on the weekends due to lower policing levels and greater numbers of inexperienced drivers, along with the intake of alcoholic beverages_._^24^ The difference found in the present study may have been due to the use of vehicles as a means of transport to go to work, which would explain the greater numbers of traffic accidents in the morning and in the middle of the week. The fact that most of the victims were alone may strengthen this hypothesis.

With regard to drowning, the fact that they occurred in the afternoon and on the weekend may denote a relationship between this kind of accident and leisure activities practiced by many families on the weekends. This result has also been pointed out by other researchers.^25^ In the case of Cuiabá, with its high temperatures and great number of rivers, it is important to consider drowning to be a major cause of accidents. The fact that most of the victims were alone again raises the essential issue of surveillance. A study in China identified greater risk of drowning among children who swam in natural water bodies without supervision from an adult (over the age of 30 years).^26^ In view of the extensive hydrographic network surrounding Cuiabá, public prevention policies, with indication of areas presenting risks, mandatory lifeguard supervision and educational prevention activities in schools and communities are also important, along with monitoring and rescue training that teaches children and adults the resuscitation techniques that are needed in order to help victims efficiently.

Aspiration of milk during the night and in the morning, which caused the death of children less than one year of age and occurred in the presence of the parents, may be related to times when the parents are tired, sleepy or even sleeping, with consequent carelessness in relation to a child who has just suckled. Aspiration of milk was singled out as the major event among small children. Effective prevention comes from the care provided by adults^27^ in feeding sessions.

Falls occurred in the morning and in the middle of the week, and this picture is consistent with workplace accidents. Accidents at work have been considered to be worrisome and to have a great social impact in Latin American and Caribbean countries.^15^ Taking developed countries such as Denmark, with only 2.9 accidents per 100,000, as an example,^28^ the importance of policies for worker protection in its various dimensions (technical, social, economic, cultural and political) has been highlighted.^29^ In the specific case of adolescents, besides raising awareness regarding the use of personal protective equipment, the safety conditions at work should be assessed in order to be able to reduce this important public health problem that affects the economically active population at its fullest stage of professional development.

The death caused by a firearm in the afternoon and in the middle of the week, at a time when the parents were working, reveals the risk of having firearms at home, often without the child’s caregiver knowing about them, such that this person also becomes a victim of the circumstances. Since firearm injuries are in most cases fatal,^30^ it becomes vital to resume oral discussions on this issue in order to move forward on disarmament.

The environmental factors found in this study are consistent with the warnings given by other authors, who considered such accidents to be the result from interaction between behavior and environmental factors.^6^ Studies have shown that exposure to stairs, windows and swimming pools without grids and to sharp objects, among other factors, increases the risk of domestic accidents, with a high hospitalization rate,^31^ often with injuries and significant sequelae.^32^ Therefore, changing the home environment is essential, combined with behavioral changes among the family, focusing on prevention.

The chemical factors observed in this study have been acknowledged in the scientific literature as significant causes of intoxication that take many children and adolescents to emergency care worldwide.^33^ Most accidents with chemical products relate to inadequate storage (within children’s reach and stored in soft-drink bottles or food containers), along with people’s lack of knowledge in relation to these products.^34^ The chemical factors combine with the family’s behavior such that, in most cases of intoxication, the product is left within the child’s reach.^35^ This once again highlights the importance of raising the awareness of the population regarding the hazards of these products and teaching people how to handle them correctly. Manufacturers’ responsibility should also be taken into consideration, because many types of packing are attractive to children and easy to open. In this regard, special child-resistant packaging (which was initially implemented in the United States and Canada) has proven to be effective. It reduced the intoxication rate by up to 35% during the period after its deployment.^36^ In Brazil, two bills of law relating to this have been put forward: the first (PL 4841/94) makes special child-resistant packaging mandatory for medicines and hazardous household products; and the second (PL 5802/01) establishes different symbols on the packaging of cleaning products according to their degree of hazard. However, these bills are still going through the legislative procedures.^37^ Although no deaths resulted from chemical factors in the present study, reflection regarding occurrences of these events with emergency care and hospital internments is required, as shown by many other studies.^38^^,^^39^^,^^40^ Hence, environmental change is an urgent preventive measure.

The presence of certain plants in people’s backyards, seen in the present study, matches the findings from a study conducted at the Pediatric Clinic of the Pontifical Catholic University of Rio Grande do Sul (Pontifícia Universidade Católica do Rio Grande do Sul, PUCRS), in which 48.3% of the parents interviewed were growing toxic plants at home.^41^ It has been pointed out that poisoning by plants is responsible for 2% of all poisoning cases and that lack of knowledge about hazardous species lies at the root of the problem.^42^ This highlights the need for guidance regarding the toxicological characteristics of plants, including widely-used ornamental species.

The presence of animals, in turn, is of concern insofar as bites can cause significant injuries, with sequelae in various spheres: emotional (resulting from the stress of exposure), physical (determined by scars and disfigurements) and economic (due to the cost of treatment and administration of vaccine and serum for rabies prevention).^5^ There are several reasons why young children are more likely than adults to be attacked on the head: first, children’s smaller stature; second, children are more likely to lean towards or put their faces close to the dog’s head, which is perceived by dogs as a threatening posture; third, adults tend to have better defenses against attacks.^43^ Since most of the accidents with animals occur in homes, there is a need for educational action regarding care measures in relation to domestic animals (castration and vaccination, and identification of symptoms that indicate the presence of diseases, especially rabies) and measures to be taken in the event of aggression (going immediately to a healthcare clinic for treatment against rabies).

Gardens with tall grass were examined, bearing in mind that this may attract poisonous animals.^7^ Trees in the backyard may give rise to falls, which have been reported to be the main cause of emergency care, leading to serious sequelae and deficits.^5^ Keeping the grass properly under control and preventing children and adolescents from climbing trees are important measures.

Regarding cultural factors, the fact that the families were unable to say whether accidents and violence were preventable or had the belief that they were not preventable was inconsistent with the positive response given when asked whether the family’s habits favored occurrences of these events. In this respect, a gap in knowledge was observed, and this was consistent with studies in which the families were unaware of the hazards at home and did not know what each stage of children’s growth and development represented in relation to accidents.^44^ Thus, it becomes important to bear to mind the epidemiological model of the accident, in which the circumstance that generated the event comprises an etiological agent (a form of energy that violates the organic tissues), a host (the child/adolescent whose stage of development makes it possible to identify the risk) and the environment (the situation in which the accident occurs),^27^ which replaces the concept of randomness.

The fact that the families supervised their children and adolescents in activities in the water but not in other leisure activities coincides with affirmations that the level of supervision is still insufficient.^45^ Comparison between the findings relating to the families’ perceptions of supervision and the fact that most of the victims of drowning were alone at the time of the accident reveals a large gap in the families’ perceptions of the risks. In the literature, this has been considered to be a factor directly related to traumatic events.^46^ The same holds for delegation of tasks, since the child is not always mature enough to take on certain tasks.^47^

## CONCLUSION

Occurrences of accidents involve multiple causal factors ranging from the family environment to cultural and social factors. Lack of knowledge among families, non-preventive culture, habits favoring occurrences of accidents, too little surveillance of children/adolescents, unsafe domestic environments with presence of hazardous products and materials, indiscriminate delegation of tasks that are incompatible with the child or adolescent’s age, lack of structure in traffic, access to firearms, work environments without safety standards, lack of more effective laws and lack of communication are some of the challenges to be understood and overcome.

Prevention is a challenging task, in which broad intersectoral action that encourages behavioral change is necessary in several social segments and in schools (both in formal and in informal groups).

Our findings point towards a need to foster safe and healthy behavior and environments, and to monitor occurrences of accidents through intersectoral actions. The importance of structuring and consolidating the attendance provided for victims should also be highlighted, in order to prevent deaths and promote rehabilitation.

Further progress is still needed with regard to the involvement of professionals in implementing the policies that have been issued, as well as in relation to better quality of information. Intersectoral actions will also be necessary, in view of the various factors involved in occurrences of accidents.

In addition, there is still a need to incorporate the issues into the curricula of various healthcare, educational and social science courses, in order to encourage reflection and create actions that contribute towards transforming the cruel reality of accidents.

Detailed knowledge of the risk factors, within a preventive approach, is essential for enabling progress in controlling accidents. Therefore, direct action in relation to risk factors and promotion of education for children, families and society, along with priority for specific policies, is urgently needed for effective control over accidents.
